# Maternal diabetes mellitus during pregnancy as a risk factor for strabismus and refractive errors in the offspring—A large‐scale national study

**DOI:** 10.1002/ijgo.70381

**Published:** 2025-07-17

**Authors:** Nir Amitai, Tomer Kerman, Tamar Eshkoli, Ahed Imtirat, Amichai Raz, Itamar Ben Shitrit, Dor Marciano, Eyal Sheiner, Erez Tsumi

**Affiliations:** ^1^ Faculty of Health Sciences, Joyce & Irving Goldman Medical School Ben‐Gurion University of the Negev Beer‐Sheva Israel; ^2^ Clinical Research Center Soroka Medical Center Beer‐Sheva Israel; ^3^ Department of Obstetrics and Gynecology Soroka Medical Center Beer‐Sheva Israel; ^4^ Endocrinology Unit Soroka Medical Center Beer‐Sheva Israel; ^5^ Department of Ophthalmology, Soroka Medical Center Ben‐Gurion University of the Negev Beer‐Sheva Israel

**Keywords:** long‐term ophthalmic outcomes, maternal diabetes, maternal glycemic control, ocular morbidity, pediatric ophthalmology, pregnancy, refractive errors, strabismus

## Abstract

**Objective:**

To investigate whether maternal diabetes during pregnancy is associated with increased risk of strabismus and refractive errors (RE) in their offspring.

**Methods:**

This retrospective cohort study utilized electronic medical records from Clalit Health Services (CHS) in Israel, 2001–2023. Births were categorized by maternal diabetes type (type 1, type 2, or gestational) or no diabetes. Mixed models compared demographics and ocular conditions, including RE and strabismus, while generalized estimating equations calculated odds ratios adjusting for confounders.

**Results:**

A total of 505 928 maternal and offspring records were analyzed. Strabismus prevalence was 496 (2.0%) in children of diabetic mothers versus 7346 (1.5%) in those of non‐diabetic mothers (odds ratio [OR] 1.29, 95% confidence interval [CI]: 1.18–1.42), and remained significant after adjustment (OR 1.40, 95% CI: 1.27–1.55). In children of mothers with HbA1c >6.5%, strabismus prevalence was 49 (2.9%) compared to 7793 (1.5%) with HbA1c ≤6.5% (adjusted OR 1.78, 95% CI: 1.30–2.43). RE prevalence was 753 (3%) among offspring of diabetic mothers versus 10 785 (2.2%) among non‐diabetic mothers (OR 1.34, 95% CI: 1.24–1.44), with maternal diabetes remaining significantly associated with RE after adjustment (OR 1.61, 95% CI: 1.48–1.74). In the HbA1c >6.5% group, RE prevalence was 71 (4.2%) versus 11 467 (2.3%) with normal HbA1c (adjusted OR 1.97, 95% CI: 1.54–2.53).

**Conclusion:**

Maternal diabetes during pregnancy is associated with strabismus and RE in the offspring, with higher risk with high HbA1c levels.

## 1 INTRODUCTION

In 2021, an estimated 527 million individuals globally were affected by diabetes, with the number projected to increase to over 1.31 billion by 2050.^1^ Diabetes during pregnancy, encompassing both pregestational diabetes (DM1 and DM2) and gestational diabetes mellitus (GDM), is increasingly prevalent, particularly in Western countries.^1^ Recent data suggest that 5.2% to 5.6% of pregnancies in the USA are complicated by GDM, with an additional 0.8% involving pre‐existing diabetes.^2^ Factors such as rising obesity rates and advanced maternal age in developed nations are expected to further elevate these rates. Recent research also highlights that maternal diabetes is associated with long‐term health complications, including cardiovascular disease, renal dysfunction, endocrine disorders, and an elevated risk of certain cancers.^3^, ^4^

The effects of maternal diabetes extend to the fetus, resulting in higher incidences of macrosomia, birth injuries, and neonatal complications, such as hemoconcentration and hypoglycemia.^5^ Studies further indicate significant long‐term effects on offspring, that may manifest years after birth, including serious health and ophthalmic morbidities.^6^

Strabismus, a common childhood eye disorder marked by misaligned eyes, can lead to vision loss from amblyopia and reduced depth perception. It also affects self‐image and social interactions, impacting overall quality of life. Many affected children require therapeutic interventions ranging from non‐surgical therapies to surgery to correct eye alignment.^7^, ^8^

The risk factors for strabismus are multifactorial and include both individual and maternal influences, such as RE, familial predisposition, prematurity, maternal smoking, and hypertensive disorders during pregnancy.^9^, ^10^

Risk factors for RE are numerous and include genetic, environmental, demographic, and medical factors.^11^ Previous studies have identified diabetes mellitus as a significant risk factor for RE, particularly for conditions such as myopia and astigmatism.^12^ RE and strabismus are closely linked, with myopia associated with exotropia and hyperopia with esotropia. Studies show that different types of RE significantly increase the risk of ocular misalignment, particularly in children.^13^

While a possible association between maternal diabetes and RE in offspring has been documented, the relationship between maternal diabetes and strabismus has not been thoroughly investigated.^12^, ^14^ To date, only one observational, single‐center study with a limited sample size, has explored this potential association, and it did not find a significant correlation between maternal diabetes and strabismus, in contrast to the established link with RE.^15^ This paucity of research highlights a notable gap in the literature regarding the potential ocular consequences of maternal diabetes on their offspring beyond RE.

The present study examined the association between maternal diabetes during pregnancy and the occurrence of strabismus and RE in their offspring.

## 2 MATERIALS AND METHODS

### 2.1 Data collection

This retrospective cohort study was conducted using the Clalit Health Services (CHS) data sharing platform, powered by MDClone (https://www.mdclone.com). This platform utilizes advanced algorithms to deidentify and extract data from electronic medical records, ensuring both data integrity and patient privacy.

### 2.2 Study population

The study analyzed medical and birth records of patients registered with CHS from January 1, 2010 to December 31, 2022. CHS, the largest health maintenance organization in Israel, provides medical services to 4.8 million individuals, representing 51% of the Israeli population. Patients were followed until the end of 2024. The year 2022 was chosen as the upper limit for inclusion to ensure at least two years of follow‐up. The population of Israel is ethnically diverse, comprising Arab, Jewish and other populations, and includes various socioeconomic and health characteristics.^16^

Inclusion criteria were records of live births from CHS for infants born during the study period to mothers also enrolled in CHS, initially totaling 518 759 records. Exclusion criteria consisted of infants with less than six months of documented follow‐up in CHS during their first year of life (13 316 infants), and 515 diagnosed with Down syndrome (ICD‐9 code: 758.0). The rationale for excluding infants with Down syndrome was to eliminate potential confounding factors associated with genetic conditions.^17^ Ultimately, 13 831 records were excluded, leaving a final study cohort of 505 928 infants.

### 2.3 Variable definitions

Maternal diabetes during pregnancy—including type 1, type 2, and gestational diabetes—was the independent variable. The primary outcome was strabismus prevalence, with secondary outcomes including RE, retinopathy of prematurity (ROP), and congenital ocular hypoplastic disease (COHD), all defined by ICD‐9 codes from the CHS database (Table S1). Diagnoses were included regardless of whether they were made during hospital admission or outpatient visits, and no additional routine examinations were performed specifically for the purposes of this study. Diabetes was identified by ICD‐9 codes or diabetes medication prescriptions (insulin, sulfonylureas, or metformin) within 9 months before delivery and classified as DM1, DM2, or GDM, with medications verified by ATC‐5 codes (Table S2). Additional demographic and clinical data, including ethnicity, socioeconomic status, maternal age, parity, birth weight, gestational age, and HbA1C levels (from 18 to 6 months before delivery), were also collected. Socioeconomic status was assigned using a municipality‐level score (1–255) based on 14 factors by the Israeli Central Bureau of Statistics. Municipalities were then grouped into clusters (1–10) and classified as low (1–3), medium (4–6), or high (7–10).^18^

### 2.4 Statistical analysis

Descriptive statistics were applied to characterize the study population, including baseline demographic and clinical characteristics. Continuous variables are presented as means, medians, standard deviations, interquartile ranges (IQR) and ranges, while categorical variables are described in terms of frequencies and percentages. Continuous variables were compared using *t*‐test for normally distributed data, and Mann–Whitney test in cases of non‐normally distributed data. Categorical variables were compared using Chi‐square test, with Fisher exact test applied when necessary. Odds of all ocular conditions were assessed using generalized estimating equations to estimate the odds ratios (OR) for infants born to mothers with diabetes relative to those without. Models were adjusted for maternal age, ethnicity, socioeconomic status, gestational week, infant sex, year of birth, multiple gestation, follow up duration and birth weight.^13^, ^19^ All statistical tests were two‐tailed with a significance threshold set at *P* less than 0.05. Analyses were performed using R software version 3.6.1.

#### Severity analysis

A first severity analysis was conducted on a selected population from the initial cohort, consisting only of infants born to mothers with diabetes. Disease severity was inferred from the type of treatment administered, dividing the population into those treated pharmacologically versus not. The former was further subdivided based on the medications used during pregnancy: insulin, sulfonylurea or metformin. A second severity analysis employed the latest available maternal HbA1C values 6 to 18 months prior to delivery as a severity index of glycemic control.

## 3 RESULTS

This study analyzed medical and birth records of 505 928 live births among 276 821 women, including 25 252 births to 16 798 mothers with diabetes during pregnancy. Of these, 982 mothers were diagnosed with DM1, 4531 with DM2, and 16 385 with GDM.

Table 1 presents comprehensive demographic and clinical profiles, revealing significant differences between the groups with diabetes and those without diabetes. The mean maternal age was lower in the non‐diabetic group (30.4 ± 5.5 years, IQR: 26.3–34.3) compared to the diabetic group (33.5 ± 5.2 years, IQR: 29.8–37.2; *P* < 0.001). Maternal parity differed between the groups (*P* < 0.001). Among non‐diabetic mothers, 135 735 (40%) were in their first pregnancy, 171 532 (51%) had two to four, and 34 025 (9.3%) had five or more, compared to 7101 (36%), 10 252 (52%), and 2366 (12%), respectively, in the group with diabetes. Infants of non‐diabetic mothers weighed an average of 3185 ± 530 g, IQR: 2900–3520, compared to 3224 ± 576 g, IQR: 2910–3586 for the group with diabetes (*P* < 0.001). Ethnic distribution revealed a lower proportion of mothers of Arabic ethnicity in the diabetic group, 5342 (21%), compared to the non‐diabetic group 120 298 (25%; *P* < 0.001). Socioeconomic status also varied, with high, medium, and low categories distributed as 84 138 (20%), 228 050 (54%), and 113 666 (27%) in the non‐diabetic group, and 4371 (19%), 13 425 (59%), and 4999 (22%) in the group with diabetes, respectively (*P* < 0.001).

**TABLE 1:** Demographic and clinical characteristics of the study groups.

Characteristic	Overall, *N* = 505 928 (276 821 mothers)	Births without maternal DM, *n* = 480 676 (264 732 mothers)	Births with maternal DM, *n* = 25 252 (16 798 mothers)	*P* value
Maternal age at delivery				**<0.001**
Mean ± SD (*n*)	30.6 ± 5.6 (505 720)	30.4 ± 5.5 (480 503)	33.5 ± 5.2 (25 217)	
Median (IQR)	30.5 (26.5, 34.5)	30.3 (26.3, 34.3)	33.6 (29.8, 37.2)	
Range	15.2, 50.0	15.2, 50.0	16.4, 50.0	
Maternal parity, *n* (%)				**<0.001**
1	142 836 (40%)	135 735 (40%)	7101 (36%)	
2–4	181 784 (51%)	171 532 (51%)	10 252 (52%)	
5+	34 025 (9.5%)	31 659 (9.3%)	2366 (12%)	
Maternal BMI, mean ± SD (*n*)	25.7 ± 44.2 (396 280)	25.5 ± 45.6 (372 537)	29.7 ± 6.2 (23 743)	**<0.001**
Ethnicity, *n* (%)				**<0.001**
Jewish	373 611 (74%)	354 116 (74%)	19 495 (77%)	
Arabic/Bedouin	125 640 (25%)	120 298 (25%)	5342 (21%)	
Other	6677 (1.3%)	6262 (1.3%)	415 (1.6%)	
Socioeconomic score, three‐level scale, *n* (%)				**<0.001**
High	88 509 (20%)	84 138 (20%)	4371 (19%)	
Medium	241 475 (54%)	228 050 (54%)	13 425 (59%)	
Low	118 665 (26%)	113 666 (27%)	4999 (22%)	
Offspring male sex	260 020 (51%)	246 853 (51%)	13 167 (52%)	**0.015**
Birth weight				**<0.001**
Mean ± SD (*n*)	3187 ± 532 (505 721)	3185 ± 530 (480 481)	3224 ± 576 (25 240)	
Median (IQR)	3220 (2900, 3525)	3218 (2900, 3520)	3255 (2910, 3586)	
Range	500, 5300	500, 5300	500, 5266	
Pre‐term birth, *n* (%)	39 968 (7.9%)	36 977 (7.7%)	2991 (12%)	**<0.001**
Multiple gestation, *n* (%)	21 777 (4.3%)	20 387 (4.2%)	1390 (5.5%)	**<0.001**

*Note*: BMI, calculated as weight in kilograms divided by the square of height in meters. Highlight values that are statistically significant, based on a *P*‐value threshold of < 0.05.

Abbreviations: BMI, body mass index; DM, diabetes mellitus; IQR, interquartile range; SD, standard deviation.

Table 2 presents incidence rates of ocular conditions in relation to type of diabetes. The incidence of strabismus among the offspring of mothers without diabetes (*n* = 7346, 1.5%) was lower compared to all diabetes types (*n* = 496, 2.0%, *P* < 0.001), DM1 (*n* = 36, 2.3%, *P* = 0.014), DM2 (*n* = 133, 2.0%, *P* = 0.005), and GDM (*n* = 483, 2.0%, *P* < 0.001). Similarly, RE prevalence in the offspring of mothers without diabetes (*n* = 10 785, 2.2%) was lower than those with diabetic mothers (*n* = 753, 3%, *P* < 0.001), particularly among those with DM1 (*n* = 52, 3.3%, *P* = 0.004). An increase was noted in the DM2 group (*n* = 178, 2.6%, *P* = 0.042), with GDM also showing a significant increase in RE (*n* = 741, 3.0%, *P* < 0.001). No significant differences were found in incidence of COHD and ROP between diabetic and non‐diabetic groups.

**TABLE 2:** Incidence of ocular conditions in relation to type of diabetes.

Ocular condition	Any DM			DM type 1			DM type 2			Gestational DM		
	No *n* = 480 676	Yes *n* = 25 252	*P* value	No *n* = 480 676	Yes *n* = 1569	*P* value	No *n* = 480 676	Yes *n* = 6815	*P* value	No *n* = 480 676	Yes *n* = 24 643	*P* value
Congenital ocular hypoplastic disease, *n* (%)	1677 (0.3)	97 (0.4)	0.356	1677 (0.3)	7 (0.4)	0.514	1677 (0.3)	18 (0.3)	0.238	1677 (0.3)	96 (0.4)	0.292
Retinopathy of prematurity, *n* (%)	558 (0.1)	24 (<0.1)	0.336	558 (0.1)	2 (0.1)	0.705	558 (0.1)	5 (<0.1)	0.303	558 (0.1)	22 (<0.1)	0.225
Refractive error, *n* (%)	10 785 (2.2)	753 (3.0)	<0.001	10 785 (2.2)	52 (3.3)	0.004	10 785 (2.2)	178 (2.6)	0.042	10 785 (2.2)	741 (3.0)	<0.001
Strabismus, *n* (%)	7346 (1.5)	496 (2.0)	<0.001	7346 (1.5)	36 (2.3)	0.014	7346 (1.5)	133 (2.0)	0.005	7346 (1.5)	483 (2.0)	<0.001

Multivariate GEE analysis, adjusted for maternal age, gestational week, gender, ethnicity, socioeconomic status, birth weight, year of birth, multiple gestation and follow‐up duration, showed significant associations between maternal diabetes and ocular conditions (Table 3). Strabismus had a crude OR of 1.29 (95% CI: 1.18, 1.42) and an adjusted OR of 1.40 (95% CI: 1.27, 1.55). RE showed a crude OR of 1.34 (95% CI: 1.24, 1.44) and an adjusted OR of 1.61 (95% CI: 1.48, 1.74). No significant associations were observed for ROP or COHD.

**TABLE 3:** Maternal diabetes, generalized estimating equations regression model multivariate analysis.

Characteristic	Crude model			Adjusted model		
	Odds ratio	95% CI	*P* value	Odds ratio	95% CI	*P* value
Retinopathy of prematurity	0.82	0.52, 1.25	0.356	0.91	0.54, 1.55	0.737
Congenital ocular hypoplastic disease	1.10	0.90, 1.35	0.361	1.17	0.94, 1.45	0.167
Refractive error	1.34	1.24, 1.44	<0.001	1.61	1.48, 1.74	<0.001
Strabismus	1.29	1.18, 1.42	<0.001	1.40	1.27, 1.55	<0.001

*Note*: Adjusted for maternal age, gestational week, infant sex, ethnicity, socioeconomic status, birth weight, year of birth, multiple gestation and follow‐up duration.

Abbreviation: CI, confidence interval.

Table 4 compares ocular condition incidence in children of diabetic mothers who received pharmacologic treatment versus those who did not. Strabismus rates were higher in treated groups overall (*n* = 150, 2.2% vs. *n* = 346, 1.9%; *P* = 0.084), particularly in the insulin (*n* = 109, 2.4%; *P* = 0.024) and sulfonylurea (*n* = 29, 2.9%; *P* = 0.026) subgroups, with no difference in the metformin group (*n* = 53, 1.7%; *P* = 0.489). RE was more common in treated mothers (*n* = 238, 3.5% vs. *n* = 515, 2.8%; *P* = 0.003), especially with insulin (*n* = 173, 3.8%; *P* < 0.001) and sulfonylurea (*n* = 52, 5.1%; *P* < 0.001), but lower with metformin (*n* = 70, 2.2%; *P* = 0.078). No significant differences were found for COHD and ROP.

**TABLE 4:** Diabetes pharmacologic treatment.

Characteristic	Pharmacologic			Insulin			Sulfonylurea			Metformin		
	No *n* = 18 474	Yes *n* = 6778	*P* value	No *n* = 18 474	Yes *n* = 4558	*P* value	No *n* = 18 474	Yes *n* = 1013	*P* value	No *n* = 18 474	Yes *n* = 3131	*P* value
Congenital ocular hypoplastic disease, *n* (%)	65 (0.4)	32 (0.5)	0.171	65 (0.4)	21 (0.5)	0.280	65 (0.4)	5 (0.5)	0.414	65 (0.4)	14 (0.4)	0.414
Retinopathy of prematurity, *n* (%)	18 (<0.1)	6 (<0.1)	0.839	18 (<0.1)	2 (<0.1)	0.401	18 (<0.1)	2 (0.2)	0.279	18 (<0.1)	4 (0.1)	0.549
Refractive error, *n* (%)	515 (2.8)	238 (3.5)	**0.003**	515 (2.8)	173 (3.8)	**<0.001**	515 (2.8)	52 (5.1)	**<0.001**	515 (2.8)	70 (2.2)	**0.078**
Strabismus, *n* (%)	346 (1.9)	150 (2.2)	0.084	346 (1.9)	109 (2.4)	**0.024**	346 (1.9)	29 (2.9)	**0.026**	346 (1.9)	53 (1.7)	0.489

*Note*: Highlight values that are statistically significant, based on a *P*‐value threshold of < 0.05.

Table 5 shows that children of mothers with HbA1c >6.5% had higher strabismus (*n* = 49, 2.9% vs. *n* = 7793, 1.5%; *P* < 0.001) and RE (*n* = 71, 4.2% vs. *n* = 11 467, 2.3%; *P* < 0.001) rates, with no differences for COHD or ROP. The multivariate analysis in Table 6 confirmed significant associations for strabismus (adjusted OR 1.78, 95% CI: 1.30–2.43) and RE (adjusted OR 1.97, 95% CI: 1.54–2.53), while ROP and COHD remained unassociated.

**TABLE 5:** Maternal glycemic control (HbA1C).

Characteristic	Overall, *N* = 505 928	HbA1C < 6.5, *N* = 504 219	HbA1C ≥ 6.5, *N* = 1709	*P* value
Congenital ocular hypoplastic disease, *n* (%)	1774 (0.4%)	1768 (0.4%)	6 (0.4%)	>0.9
Retinopathy of prematurity, *n* (%)	582 (0.1%)	578 (0.1%)	4 (0.2%)	0.14
Refractive error, *n* (%)	11 538 (2.3%)	11 467 (2.3%)	71 (4.2%)	**<0.001**
Strabismus, *n* (%)	7842 (1.6%)	7793 (1.5%)	49 (2.9%)	**<0.001**

*Note*: Highlight values that are statistically significant, based on a *P*‐value threshold of < 0.05.

**TABLE 6:** Maternal glycemic control, generalized estimating equations regression model multivariate analysis (HbA1C).

Characteristic	Crude model			Adjusted model		
	Odds ratio	95% CI	*P* value	Odds ratio	95% CI	*P* value
Retinopathy of prematurity	2.04	0.61, 6.82	0.245	1.27	0.31, 5.18	0.735
Congenital ocular hypoplastic disease	1.00	0.45, 2.23	0.998	1.04	0.47, 2.32	0.919
Refractive error	1.86	1.47, 2.36	<0.001	1.97	1.54, 2.53	<0.001
Strabismus	1.88	1.41, 2.50	<0.001	1.78	1.30, 2.43	<0.001

Abbreviation: CI, confidence interval.

## 4 DISCUSSION

Recent research has increasingly focused on the impact of maternal diabetes on morbidities among their offspring, including ocular conditions. A correlation has been established between maternal diabetes and various ocular disorders, and long‐term ocular morbidities.^6^, ^12^ The current large‐scale cohort study analyzed 505 928 medical records of maternal patients and their offspring. The findings revealed a significant association between maternal diabetes and the occurrence of strabismus and RE in the offspring.

The relationship between maternal diabetes and strabismus remains inadequately explored. Although several studies have demonstrated an association between maternal smoking during pregnancy and strabismus in offspring, highlighting the impact of the intrauterine environment on strabismus development, research on the link between maternal diabetes and strabismus is limited.^20^ A study by Alvarez‐Bulnes et al., directly investigated this association. Their research, which observed 350 children, reported no significant correlation between maternal diabetes and strabismus.^15^ However, this study was constrained by a limited sample size and single‐center design, potentially affecting the generalizability and statistical power of the findings.

Our investigation utilized comprehensive medical records from CHS, Israel's largest healthcare provider. After adjusting for multiple factors the analysis revealed a 40% increase in incidence of strabismus among offspring of mothers with diabetes. Moreover, when considering maternal glycemic control, the study demonstrated a 78% increase in strabismus prevalence among offspring of mothers with uncontrolled diabetes, as evidenced by elevated HbA1C levels. These findings provide support for the potential influence of maternal diabetes, particularly hyperglycemic states, on the development of strabismus in their offspring.

The association between maternal diabetes and RE in offspring has been increasingly supported by recent research. A comprehensive, nationwide registry‐based cohort study by Du et al., involving 2 470 580 individuals, found a 39% increased association with high RE in children born to diabetic mothers in Denmark.^12^ Similarly, a prospective cohort study by Xu et al., examining 301 individuals born 2015–2017, reported a 171% increased risk of high RE in offspring prenatally exposed to maternal diabetes.^14^

Our findings support recent literature, revealing 61% higher odds of RE in offspring of diabetic mothers, with an even more pronounced 97% increase in odds for children of mothers with uncontrolled diabetes, as indicated by the HbA1C values. The exact mechanisms underlying this association are not fully understood. However, previous studies proposed several pathophysiological explanations. One hypothesis suggests that embryonic hyperglycemia may lead to a breakdown of the blood‐ocular barrier endothelial system. Additionally, the hyperglycemic environment is thought to enhance oxidative stress and trigger inflammatory responses, which could contribute to the development of ocular abnormalities in offspring of diabetic mothers.

The association between maternal diabetes during pregnancy and ROP in offspring is a subject of ongoing debate in the scientific literature. While several studies identified maternal diabetes as a potential risk factor for ROP in low birth weight infants,^21^ conflicting evidence exists. A comprehensive meta‐analysis conducted by Razak et al., which examined nine observational studies encompassing almost 1.2 million preterm infants, found no significant association between maternal diabetes and ROP.^22^ Our findings align with this meta‐analysis, as we observed no significant relationship between maternal diabetes and the development of ROP in offspring.

The body of literature examining the relationship between maternal diabetes and ocular hypoplastic disorders in offspring is sparse. Although previous research suggested a potential link between maternal DM1 and superior segmental optic nerve hypoplasia,^23^ our comprehensive, population‐based investigation failed to corroborate this association. This discrepancy highlights the need for further research to elucidate the complex interplay between maternal metabolic conditions and fetal ocular development.

The association between in utero exposure to elevated glucose levels and several ophthalmic morbidities may be mediated through various mechanisms. Research has demonstrated that hyperglycemic conditions can modulate the activity of multiple transcription factors.^24^ While the embryonic phase represents a critical window in which such disruptions could lead to structural anomalies—particularly in cases of pre‐existing diabetes—the observed associations with gestational diabetes suggest that hyperglycemia in later stages of pregnancy may also play a role. This may occur through effects on neuronal maturation, vascular development, or other post‐embryonic pathways. These findings highlight the need for further research into both the timing and mechanisms by which maternal hyperglycemia may affect ocular outcomes in offspring.

## 5 STRENGTHS AND LIMITATIONS

The large sample size of the present study and extended data collection provided robust statistical power and likely mitigated selection bias, enhancing generalizability. However, its retrospective design prevents causal inferences, and the lack of physical examinations limited our ability to determine the laterality of strabismus, RE, or other ocular conditions.

## 6 CONCLUSION

Maternal diabetes during pregnancy is associated with higher rates of strabismus and RE in their offspring. This risk is even higher among infants born to mothers with uncontrolled diabetes.

## AUTHOR CONTRIBUTIONS

**Nir Amitai**: Led the writing of the article, performed data analysis and data extraction, contributed to the design of the study, wrote the initial draft and contributed to revisions. Approved the final manuscript and is accountable for all aspects of the work. **Tomer Kerman**: Interpreted data findings and assisted in study design considerations, contributed to drafting and revising sections related to data interpretation. Reviewed and approved the final version, accepting responsibility for its content. **Tamar Eshkoli**: Provided obstetrics and gynecology (OBGYN) expertise and critical input to refine methodology and interpretation. Critically reviewed the manuscript for important intellectual content in the OBGYN domain. Approved the final version and is accountable for the OBGYN‐related content. **Ahed Imtirat:** Contributed ophthalmic expertise, refining the study design and interpretation of ophthalmic data. Provided critical revisions and input specific to ophthalmology. Approved the final manuscript and remains responsible for ophthalmic accuracy. **Amichai Raz**: Conducted literature review, helped plan the research, and refined the methodology. Provided critical feedback and revisions, ensuring methodological soundness. Gave final approval and is accountable for the integrity of the research planning. **Itamar Ben Shitrit**: Participated in writing and editing the manuscript, contributed to the research design. Assisted in drafting and refining the text. Approved the final version, agreeing to be accountable for its content. **Dor Marciano**: Provided additional OBGYN inputs and helped interpret clinical data from an obstetric perspective. Reviewed and refined relevant sections for clinical accuracy. Approved the final version, ensuring OBGYN content is accurate. **Eyal Sheiner**: Oversaw the study, offered critical revisions, and provided guidance on study design and methodology. Reviewed the manuscript comprehensively, refining it for intellectual content. Approved the final version and is accountable for the overall study integrity. **Erez Tsumi**: Interpreted the results, supervised writing and planning, and provided strategic oversight of the study. Reviewed and revised the manuscript, ensuring cohesion and accuracy. Approved the final version, accepting overall responsibility for the work. Each author confirms they have read and approved the final manuscript and agrees to be accountable for all aspects of the work, in accordance with ICMJE guidelines.

## FUNDING INFORMATION

The authors received no specific funding for this work.

## CONFLICT OF INTEREST STATEMENT

The authors have no conflicts of interest.

## Supporting information


Data S1.

## Data Availability

The data that support the findings of this study are available on request from the corresponding author. The data are not publicly available due to privacy or ethical restrictions.

## References

[1] Ong KL , Stafford LK , McLaughlin SA , et al. Global, regional, and national burden of diabetes from 1990 to 2021, with projections of prevalence to 2050: a systematic analysis for the global burden of disease study 2021. Lancet. 2023;402:203‐234. doi:10.1016/S0140-6736(23)01301-6 37356446 PMC10364581

[2] Deputy NP , Kim SY , Conrey EJ , Bullard KM . Prevalence and changes in preexisting diabetes and gestational diabetes among women who had a live birth—United States, 2012–2016. MMWR Morb Mortal Wkly Rep. 2018;67:1201‐1207. doi:10.15585/mmwr.mm6743a2 PMC6319799 30383743

[3] Fuchs O , Sheiner E , Meirovitz M , Davidson E , Sergienko R , Kessous R . The association between a history of gestational diabetes mellitus and future risk for female malignancies. Arch Gynecol Obstet. 2017;295:731‐736. doi:10.1007/s00404-016-4275-7 28035489

[4] Beharier O , Shoham‐Vardi I , Pariente G , et al. Gestational diabetes mellitus is a significant risk factor for long‐term maternal renal disease. J Clin Endocrinol Metab. 2015;100:1412‐1416. doi:10.1210/jc.2014-4474 25668200

[5] Persson B , Hanson U . Neonatal morbidities in gestational diabetes mellitus. Diabetes Care. 1998;21:B79‐B84. 9704232

[6] Walter E , Tsumi E , Wainstock T , Spiegel E , Sheiner E . Maternal gestational diabetes mellitus: is it associated with long‐term pediatric ophthalmic morbidity of the offspring? J Matern Fetal Neonatal Med. 2019;32:2529‐2538. doi:10.1080/14767058.2018.1439918 29429374

[7] Menon V , Saha J , Tandon R , Mehta M , Khokhar S . Study of the psychosocial aspects of strabismus. J Pediatr Ophthalmol Strabismus. 2002;39:203‐208. doi:10.3928/0191-3913-20020701-07 12148552

[8] Jin K , Aboobakar IF , Whitman MC , Oke I . Mental health conditions associated with strabismus in a diverse cohort of US adults. JAMA Ophthalmol. 2024;142:472‐475. doi:10.1001/jamaophthalmol.2024.0540 38573646 PMC11099685

[9] Zhu H , You X , Jing Y , et al. Maternal hypertensive disorder in pregnancy and childhood strabismus in offspring. JAMA Netw Open. 2024;7:e2423946. doi:10.1001/jamanetworkopen.2024.23946 39037813 PMC11265127

[10] Zhang XJ , Lau YH , Wang YM , et al. Prevalence of strabismus and its risk factors among school aged children: the Hong Kong Children Eye Study. Sci Rep. 2021;11:13820. doi:10.1038/s41598-021-93131-w 34226578 PMC8257606

[11] Wang M , Ma J , Pan L , et al. Prevalence of and risk factors for refractive error: a cross‐sectional study in Han and Mongolian adults aged 40–80 years in Inner Mongolia, China. Eye. 2019;33:1722‐1732. doi:10.1038/s41433-019-0469-0 31160702 PMC7002754

[12] Du J , Li J , Liu X , et al. Association of maternal diabetes during pregnancy with high refractive error in offspring: a nationwide population‐based cohort study. Diabetologia. 2021;64:2466‐2477. doi:10.1007/s00125-021-05526-z 34401952

[13] Lee SH , Jung SJ , Ohn Y‐H , Chang JH . Association between refractive errors and horizontal strabismus: the Korea National Health and Nutrition Examination Survey. J AAPOS. 2021;25:340.e1‐340.e7. doi:10.1016/j.jaapos.2021.07.012 34752909

[14] Xu Q , Zhang F , Li J , et al. Association of maternal diabetes during pregnancy with visual acuity development in offspring: a prospective cohort study. Acta Diabetol. 2022;59:1461‐1468. doi:10.1007/s00592-022-01933-9 35941247

[15] Alvarez‐Bulnes O , Monés‐Llivina A , Cavero‐Roig L , et al. Ophthalmic pathology in the offspring of pregnant women with gestational diabetes mellitus. Matern Child Health J. 2020;24:524‐529. doi:10.1007/s10995-020-02887-6 31997119

[16] Gabbay J , Walter E , Kerman T , et al. Hypoperfusion states could increase the risk of non‐arteritic anterior ischemic optic neuropathy. PLoS One. 2024;19:e0313098. doi:10.1371/journal.pone.0313098 39585890 PMC11588264

[17] Cregg M , Woodhouse JM , Stewart RE , et al. Development of refractive error and strabismus in children with down syndrome. Invest Ophthalmol Vis Sci. 2003;44:1023‐1030. doi:10.1167/iovs.01-0131 12601024

[18] Central Bureau of Statistics . Statistical abstract of Israel, no. 69. 2018.

[19] Tang SM , Chan RYT , Bin Lin S , et al. Refractive errors and concomitant strabismus: a systematic review and meta‐analysis. Sci Rep. 2016;6:35177. doi:10.1038/srep35177 27731389 PMC5059633

[20] Yang Y , Wang C , Gan Y , et al. Maternal smoking during pregnancy and the risk of strabismus in offspring: a meta‐analysis. Acta Ophthalmol (Oxford, England). 2019;97:353‐363. doi:10.1111/aos.13953 30402966

[21] Tunay ZÖ , Özdemir Ö , Acar DE , Öztuna D , Uraş N . Maternal diabetes as an independent risk factor for retinopathy of prematurity in infants with birth weight of 1500 g or more. Am J Ophthalmol. 2016;168:201‐206. doi:10.1016/j.ajo.2016.05.022 27287819

[22] Razak A , Faden M . Association of maternal diabetes mellitus with preterm infant outcomes: a systematic review and meta‐analysis. Arch Dis Child Fetal Neonatal Ed. 2021;106:271‐277. doi:10.1136/archdischild-2020-320054 33172874

[23] Landau K , Bajka JD , Kirchschläger BM . Topless optic disks in children of mothers with type I diabetes mellitus. Am J Ophthalmol. 1998;125:605‐611. doi:10.1016/S0002-9394(98)00016-6 9625543

[24] Xi G , Wai C , White MF , Clemmons DR . Down‐regulation of insulin receptor substrate 1 during hyperglycemia induces vascular smooth muscle cell dedifferentiation. J Biol Chem. 2017;292:2009‐2020. doi:10.1074/jbc.M116.758987 28003360 PMC5290970

