# Supplementary material for: Maternal diabetes mellitus during pregnancy as a risk factor for strabismus and refractive errors in the offspring—A large‐scale national study
Source: Int J Gynaecol Obstet. 2025 Jul 17;172(1):565–72. doi: 10.1002/ijgo.70381 (PMC12724061; doi:10.1002/ijgo.70381)
Supplement: Supplementary file 1 — Data S1. [file IJGO-172-565-s001.docx]

Supplementary table 1. List of ICD-9 codes

| **Description** | **ICD9 code** | **Disease** |
| --- | --- | --- |
| Retinopathy of prematurity, unspecified | 362.20 | **Ischemic retinal state & Retinopathy Of Prematurity (ROP)** |
| Retrolental fibroplasia | 362.21 |  |
| ROP Stage 0 | 362.22 |  |
| ROP Stage 1 | 362.23 |  |
| ROP Stage 2 | 362.24 |  |
| ROP Stage 3 | 362.25 |  |
| ROP Stage 4 | 362.26 |  |
| ROP Stage 5 | 362.27 |  |
| Retinal ischemia | 362.84 |  |
| Background retinopathy, unspecified | 362.10 |  |
| Unspecified disorder of refraction and accommodation | 367.9 | **Refractive Error** |
| Astigmatism, unspecified | 367.20 |  |
| Hypermetropia (aka. Hyperopia) | 367.0 |  |
| Myopia (aka. Nearsightedness) | 367.1 |  |
| Optic nerve hypoplasia | 377.43 | **Congenital Abnormalities and Optic Nerve Hypoplasia and other neuro-ophthalmological disease** |
| Specified congenital anomalies of optic disc | 743.57 |  |
| Congenital anomalies of eye | 743 |  |
| MICROPHLAMOS | 743.1X |  |
| BUPHTHALMUS (congenital glaucoma, newborn glaucoma) | 742.2 |  |
| Glaucoma of childhood | 365.14 |  |
| Coloboma and other anomalies of anterior segment | 743.4X |  |
| Congenital anomalies of posterior segment | 743.5X |  |
| Other specified anomalies of eye | 743.8 |  |
| Unspecified anomaly of eye | 743.9 |  |
| Other specified congenital anomalies of nervous system | 742.8 |  |
| Optic nerve pit (aka. Crater-Like hole in optic disc) | 377.22 |  |
| Other specified congenital anomalies of iris and ciliary body | 743.46 |  |
| Periventricular leukomalacia | 779.7 |  |
| Type 1 diabetes mellitus with ketoacidosis, controlled | 250.11 | **DM1** |
| Type 1 diabetes mellitus with ketoacidosis, uncontrolled | 250.13 |  |
| Type 1 diabetes mellitus with hyperosmolarity, controlled | 250.21 |  |
| Type 1 diabetes mellitus with hyperosmolarity, uncontrolled | 250.23 |  |
| Type 1 diabetes mellitus with other coma, controlled | 250.31 |  |
| Type 1 diabetes mellitus with other coma, uncontrolled | 250.33 |  |
| Type 1 diabetes mellitus with renal manifestations, controlled | 250.41 |  |
| Type 1 diabetes mellitus with renal manifestations, uncontrolled | 250.43 |  |
| Type 1 diabetes mellitus with ophthalmic manifestations, controlled | 250.51 |  |
| Type 1 diabetes mellitus with ophthalmic manifestations, uncontrolled | 250.53 |  |
| Type 1 diabetes mellitus with neurological manifestations, controlled | 250.61 |  |
| Type 1 diabetes mellitus with neurological manifestations, uncontrolled | 250.63 |  |
| Type 1 diabetes mellitus with peripheral circulatory disorders, controlled | 250.71 |  |
| Type 1 diabetes mellitus with peripheral circulatory disorders, uncontrolled | 250.73 |  |
| Type 1 diabetes mellitus with other specified manifestations, controlled | 250.81 |  |
| Type 1 diabetes mellitus with other specified manifestations, uncontrolled | 250.83 |  |
| Type 1 diabetes mellitus with unspecified complication, controlled | 250.91 |  |
| Type 1 diabetes mellitus with unspecified complication, uncontrolled | 250.93 |  |
| Diabetes mellitus without mention of complication, type II or unspecified type, not stated as uncontrolled | 250.00 | **DM2** |
| Diabetes mellitus without mention of complication, type II or unspecified type, uncontrolled | 250.02 |  |
| Diabetes with ketoacidosis, type II or unspecified type, not stated as uncontrolled | 250.10 |  |
| Diabetes with ketoacidosis, type II or unspecified type, uncontrolled | 250.12 |  |
| Diabetes with hyperosmolarity, type II or unspecified type, not stated as uncontrolled | 250.20 |  |
| Diabetes with hyperosmolarity, type II or unspecified type, uncontrolled | 250.22 |  |
| Diabetes with other coma, type II or unspecified type, not stated as uncontrolled | 250.30 |  |
| Diabetes with other coma, type II or unspecified type, uncontrolled | 250.32 |  |
| Diabetes with renal manifestations, type II or unspecified type, not stated as uncontrolled | 250.40 |  |
| Diabetes with renal manifestations, type II or unspecified type, uncontrolled | 250.42 |  |
| Diabetes with ophthalmic manifestations, type II or unspecified type, not stated as uncontrolled | 250.50 |  |
| Diabetes with ophthalmic manifestations, type II or unspecified type, uncontrolled | 250.52 |  |
| Diabetes with neurological manifestations, type II or unspecified type, not stated as uncontrolled | 250.60 |  |
| Diabetes with neurological manifestations, type II or unspecified type, uncontrolled | 250.62 |  |
| Diabetes with peripheral circulatory disorders, type II or unspecified type, not stated as uncontrolled | 250.70 |  |
| Diabetes with peripheral circulatory disorders, type II or unspecified type, uncontrolled | 250.72 |  |
| Diabetes with other specified manifestations, type II or unspecified type, not stated as uncontrolled | 250.80 |  |
| Diabetes with other specified manifestations, type II or unspecified type, uncontrolled | 250.82 |  |
| Diabetes with unspecified complication, type II or unspecified type, not stated as uncontrolled | 250.90 |  |
| Diabetes with unspecified complication, type II or unspecified type, uncontrolled | 250.92 |  |
| Diabetes mellitus complicating pregnancy, unspecified episode of care | 648.00 | **GDM** |
| Diabetes mellitus complicating pregnancy, delivered, with or without mention of antepartum condition | 648.01 |  |
| Diabetes mellitus complicating pregnancy, delivered, with mention of postpartum complication | 648.02 |  |
| Diabetes mellitus complicating pregnancy, antepartum condition or complication | 648.03 |  |
| Diabetes mellitus complicating pregnancy, postpartum condition or complication | 648.04 |  |
| Abnormal glucose tolerance complicating pregnancy, childbirth, or the puerperium, unspecified as to episode of care or not applicable | 648.80 | **Glucose intolerance during pregnancy** |
| Abnormal glucose tolerance complicating pregnancy, delivered, with or without mention of antepartum condition | 648.81 |  |
| Abnormal glucose tolerance complicating pregnancy, delivered, with mention of postpartum complication | 648.82 |  |
| Abnormal glucose tolerance during pregnancy, antepartum | 648.83 |  |
| Abnormal glucose tolerance during pregnancy, postpartum | 648.84 |  |
| Down syndrome | 758.0 | **Down syndrome** |
| Strabismus and other disorders of binocular eye movements | 378.x | **Strabismus** |

**Supplementary table 2.** List of ATC5 medication codes

| **Drug type** | **ATC3 code** | **ATC5 code** | **Drug name** |
| --- | --- | --- | --- |
| **Insulin** | A10A*** | A10AB06 | INS APIDRA SOLOSTAR |
|  |  | A10AE04 | INS BASAGLAR KWIK |
|  |  | A10AE04 | INS LANTUS SOLOSTAR |
|  |  | A10AE05 | INS LEVEMIR FLEX PEN |
|  |  | A10AB05 | INS NOVORAPID FLEX PEN |
|  |  | A10AE04 | INS TOUJEO PEN |
|  |  | A10AE06 | INS TREGLUDEC PEN |
|  |  | A10AB04 | INS HUMALOG KWIK PEN |
|  |  | A10AD05 | INS NOVOMIX 30 FLEX |
|  |  | A10AE06 | INS XULTOPHY |
| **Metformin** | A10B*** | A10BA02 | GLUCOMIN CPL |
|  |  | A10BA02 | GLUCOPHAGE TAB |
|  |  | A10BA02 | JANUET TAB |
|  |  | A10BA02 | JARDIANCE DUO |
| **Sulfonylureas** | A10BB** | A10BB12 | AMARYL TAB |
|  |  | A10BB01 | GLIBENCLAMIDE TAB |
|  |  | A10BB01 | GLIBETIC TAB |
|  |  | A10BB07 | GLUCO RITE TAB |
|  |  | A10BB12 | AMARYL TAB |
|  |  | A10BB12 | GLIMEPIRIDE TAB |
|  |  | A10BB02 | DIABETEX TAB |
| **Aspirin** | B01AC06 | B01AC06; N02BA01 | ACETYLSALICYLIC ACID TAB |
|  |  | B01AC06 | CARTIA TAB 100mg 28 |
|  |  | B01AC06 | MICROPIRIN TAB |
|  |  | B01AC06 | TEVAPIRIN TAB |
|  |  | B01AC06; N02BA01 | BUFFERED PIRIN |
|  |  | B01AC06; N02BA01 | ACETOSAL TAB |
|  |  | B01AC06 | ECOPRIN TAB |
